# Live cell imaging of mitochondria following targeted irradiation *in situ* reveals rapid and highly localized loss of membrane potential

**DOI:** 10.1038/srep46684

**Published:** 2017-04-25

**Authors:** Dietrich W. M. Walsh, Christian Siebenwirth, Christoph Greubel, Katarina Ilicic, Judith Reindl, Stefanie Girst, Giovanna Muggiolu, Marina Simon, Philippe Barberet, Hervé Seznec, Hans Zischka, Gabriele Multhoff, Thomas E. Schmid, Guenther Dollinger

**Affiliations:** 1 Universität der Bundeswehr München, Institut für Angewandte Physik und Messtechnik, D-85577 Neubiberg, Germany; 2 Klinikum rechts der Isar, Technische Universität München, D-81675 München, Germany; 3 Université de Bordeaux, Centre d’Etudes Nucléaires de Bordeaux Gradignan (CENBG), Chemin du Solarium, 33175 Gradignan, France; 4 IN2P3, CNRS, UMR5797, Centre d’Etudes Nucléaires de Bordeaux Gradignan (CENBG), Chemin du Solarium, 33175 Gradignan, France; 5 Institute of Molecular Toxicology and Pharmacology, Helmholtz Center Munich, German Research Center for Environmental Health, D-85764 Neuherberg, Germany

## Abstract

The reliance of all cell types on the mitochondrial function for survival makes mitochondria an interesting target when trying to understand their role in the cellular response to ionizing radiation. By harnessing highly focused carbon ions and protons using microbeams, we have performed *in situ* live cell imaging of the targeted irradiation of individual mitochondria stained with Tetramethyl rhodamine ethyl ester (TMRE), a cationic fluorophore which accumulates electrophoretically in polarized mitochondria. Targeted irradiation with both carbon ions and protons down to beam spots of <1 μm induced a near instant loss of mitochondrial TMRE fluorescence signal in the targeted area. The loss of TMRE after targeted irradiation represents a radiation induced change in mitochondrial membrane potential. This is the first time such mitochondrial responses have been documented *in situ* after targeted microbeam irradiation. The methods developed and the results obtained have the ability to shed new light on not just mitochondria’s response to radiation but to further elucidate a putative mechanism of radiation induced depolarization and mitochondrial response.

The detrimental effects of ionizing radiation on the human body as a whole have been studied since 1896^1^ and, after decades of work, have been linked to the formation of DNA lesions, and classified as a risk factor for the development of cancer. Ionizing radiation has also been harnessed very successfully as a tool in the treatment of cancers. However, many key questions regarding the effects of ionizing radiation on cells, cancerous and non-cancerous, still remain unanswered. The main focus of research conducted into the effects of ionizing irradiation on cells has focused on the damage to the cell nucleus and the detrimental effects this has upon the cell^2^. The prevailing dogma in radiation biology and radiotherapy is that a high enough dose of energy deposited to the nucleus will ultimately lead to the destruction of that cell. Within this “classical dogma”, the cytoplasm, the cellular environment in which the majority of cellular processes involved in the maintenance of cellular integrity take place and which makes up a large part of each cell by volume, have rarely been taken into account as the cytoplasm has been assumed to be less sensitive to radiation.

The first cytoplasmic irradiation experiments were documented in 1953 by Zirkle and Bloom^3^ and have more recently been intensively conducted using a variety of tools, including femto-second lasers^4^,^5^,^6^,^7^ and particle micro beams^8^. The overall consensus, since 1953, is still that the cytoplasm as a whole is less radio-sensitive than the nucleus^9^. Cytoplasmic irradiation has been shown to be involved in inducing bystander effects^10^ and mutation induction^11^. The question therefore remains what contribution the cytoplasmic components have in damage induction and cellular survival.

Owing to the number of constituents and the high density of proteins, the cytoplasm is a highly complicated environment to study. In the majority of mammalian, cells the cytoplasm makes up the largest volume of the cell and to investigate the cytoplasm means to investigate the response to radiation of a variety of individual organelles and biological components. In this study mitochondria have been selected to highlight the effect of targeted irradiation. Mitochondria constitute a large volume of the cytoplasm in all cell types found in the body as they are the main site of cellular energy homeostasis in normal and cancer cells.

Mitochondrial function is directly linked to mitochondrial polarization state. Intact mitochondria are polarized, i.e. they sustain a highly charged (negative inside) membrane potential for full functionality^12^. Membrane potential is a key feature of mitochondria, as the loss of the potential across the membrane is accompanied by a variety of cellular responses, including cytochrome c release, and is involved in apoptotic cell death^13^.

The majority of previous work on the response of mitochondria to radiation has been performed using lasers^4^,^5^,^6^,^7^. However, due to the nature of energy deposition of lasers, such experiments do not enable a quantification of the energy deposited in individual mitochondria and only a minor fraction of molecular species may be affected. This is where particle radiation and ion beams become invaluable radiation techniques. Focused ion beam irradiation allows for the quantification of deposited energy equally distributed over all molecular species and consequently can be linked to the effect on the mitochondria. The heavy ion microbeam SNAKE (Super conducting Nanoprobe for Applied Nuclear (Kern) physics Experiments) based in Munich and the Proton Microbeam AIFIRA (Applications Interdisciplinaires de Faisceaux d’Ions en Région Aquitaine) in Bordeaux are ideal tools to probe the radiation response to minute cellular constituents, such as mitochondria.

Tetramethyl rhodamine ethyl ester (TMRE) a cationic fluorophore, which accumulates electrophoretically in polarized mitochondria^14^ enables the assessment of mitochondrial membrane potential, and therefore mitochondrial function, allowing for changes in membrane potential to be visualized very rapidly^15^. This membrane permeable dye allows for a simple “on/off” readout of fluorescent signal accumulation in mitochondria in direct relation to the mitochondrial membrane potential ΔΨ_m_. In functional and polarized mitochondria, ΔΨ_m_ ranges between −120 to −200 mV and mitochondria accumulate the positively charged TMRE in direct relation to the negative membrane potential within the mitochondria. If ΔΨ_m_ is increasingly lost, the fluorescence signal dissipates as the mitochondrial ΔΨ_m_ increases. Live cell irradiation combined with fast online fluorescence microscopy therefore enables irradiation and imaging, with a delay of only a few seconds, for live cells under controlled cell culture conditions (temperature, medium, pH, humidity). Mitochondria vary between ~0.2–0.5 μm in width and ~0.5–10 μm in length. They are highly dynamic and, in many cases, strongly networked organelles that are in constant movement. Therefore irradiation and imaging must be performed in fast succession for any usable data to be obtained. Both SNAKE and AIFIRA beamlines enable fast and accurate data acquisition using Zeiss epifluorescence microscopes in line with the beam exit windows. The techniques enable the exploration of the response of mitochondria to highly specific energy deposition with beamspot sizes which are in the size range of the mitochondria themselves.

## Results

**Figure 1:**
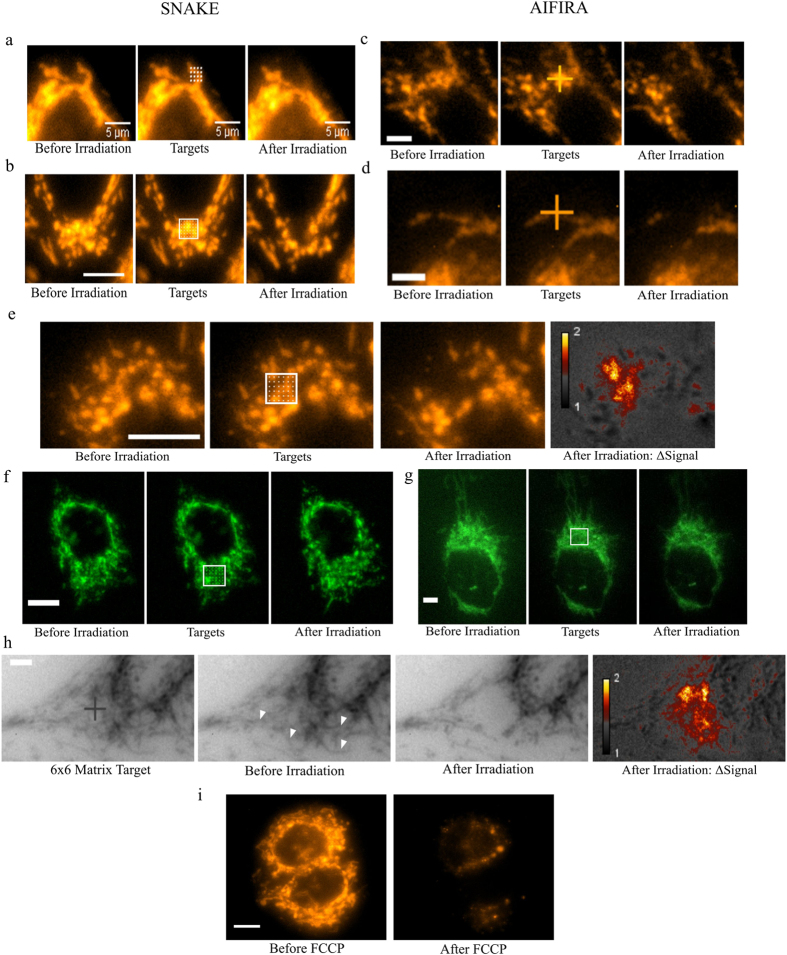
Micrographs of irradiated MCF7 and A549 cells from experiments at SNAKE (**a,b,e**) and AIFIRA (**c,d,g,h**); unless specifically stated scale bars represent 10 μm. Successive images (left to right) show pre-irradiation, target placement on pre-irradiation image and post irradiation. (**a,b**) TMRE stained cells irradiated with 100 carbon ions per point show mitochondrial depolarization (localized loss of signal) of targeted and consequently irradiated mitochondria with 4 × 4 irradiation points for MCF7 (**a**), 6 × 6 points with A549 (**b**). (**e**) Targeted irradiation performed at SNAKE. Images show pre and post irradiation with 6 × 6 matrix and 100 carbon ions per point. The pseudocolour images (**e,h**) depict the changes in signal between pre and post irradiation. Before images were divided by after images to obtain a 32 bit float image, the changes were represented in a pseudocolour look up table (Smart, Fiji). A value of 1 (gray) equates to no difference between before and after and a value of up to 2 (yellow) equates to a drop in signal intensity. (**f**) MTG stained cells do not show localized loss of signal after irradiation with 100 carbon ions per point. (**c**,**d**) Experiments performed at AIFIRA show the same loss of TMRE after irradiation with an equivalent number of protons. (**g**) HTB U2OS mito-RoGFP2 tagged cells irradiated with 3 × 3 target matrix do not show any change of mitochondria staining after irradiation with 6800 protons per point (equivalent to 200 carbon ions). (**h**) Inverted greyscale representation used to depict the irradiation (6 × 6 point matrix) and depolarization of a whole interconnected network of mitochondria up to 18 μm away from the irradiation site. Pseudocolour image (ΔSignal) represents change in signal between before and after irradiation. (**i**) Control experiment depicting mitochondria stained with TMRE before treatment with 1 μM FCCP and 10 s after. Images depict the loss of specific, TMRE signal after uncoupling of mitochondrial membrane potential and are comparable to that induced by irradiation induced damage.

TMRE accumulates electrophoretically within the cell in a concentration dependent manner that is directly related to the ΔΨ_m_ of each individual mitochondria, yielding a bright mitochondrial fluorescence signal and a less pronounced cytoplasmic background^12^. The accumulation of TMRE fluorescence in the cytoplasm and in the mitochondria is in direct relation to the membrane potential, which is maintained across the cellular and mitochondrial membranes respectively. Polarized mitochondria labelled with TMRE were visualized in both A549 and MCF7 cell lines and consequently irradiated with either arrays of irradiation points targeted to single (Fig. 1a) or clusters of mitochondria (Fig. 1b) using both 55 MeV carbon ions (SNAKE) or 3 MeV protons (AIFIRA). Each beam spot represents a counted number of carbon ions ranging from 1–100 55 MeV carbon ions (LET = 350 keV/μm) focused to <1 μm per point or, in the case of protons, 700 to 14000 protons (LET = 10 keV/μm) per point focused to <1.5 μm. To deposit the same amount of energy as 1 carbon ion applied at SNAKE, 35 protons were used at AIFIRA. The dose rate was adjusted so that the same amount of energy was deposited per second; to ensure this was the case, carbon ions were applied at a count rate of 1 kHz and protons at 35 kHz.

**Figure 2:**
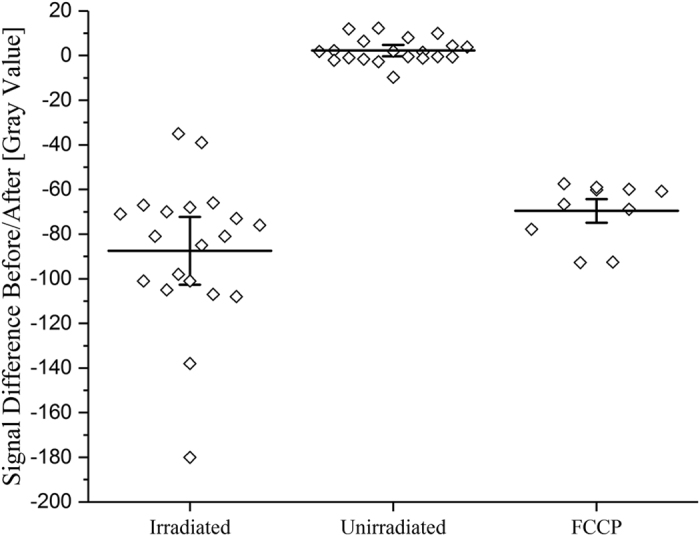
Quantification of TMRE signal before and after irradiation with carbon ions of 20 individually irradiated and unirradiated areas in 20 separate cells as well as 10 areas for FCCP control experiments. The individual points represent measurements, the horizontal lines represent the means and the error bars represent 95% CI. FCCP control experiments and the irradiated values overlap, and fall within a region normal for depolarization. The irradiation was performed using 80 ions per point in a 6 × 6 matrix. The irradiation areas were analyzed before and after irradiation by quantifying the background subtracted gray values in the micrographs before and after irradiation. The unirradiated controls consisted of mitochondria in the same irradiated cell but more than 10 μm away from the irradiated area. A paired two tailed t-test indicates a highly significant result (P < 0.001) between non-irradiated and irradiated samples, and the effect size as calculated by Cohen’s d is 3.85, making the results highly significant.

**Figure 3:**
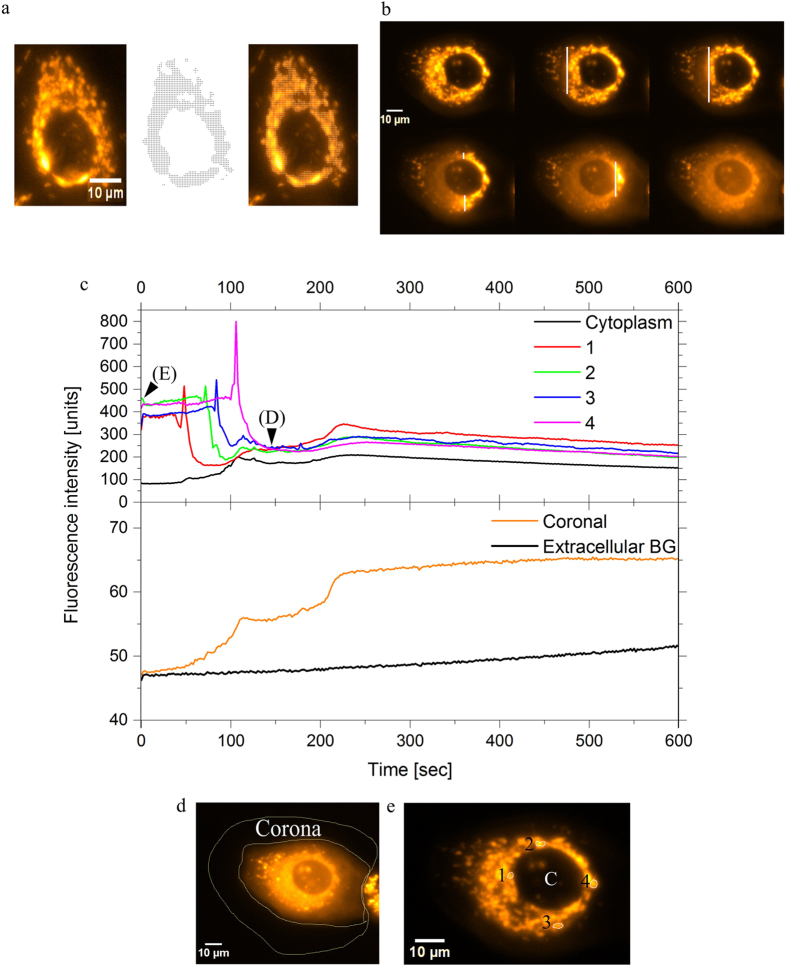
Results for MCF7 cells stained with TMRE during irradiation experiments at SNAKE are shown. (**a**) Automatic targeting macro “AutoTarget” as used for recognition and irradiation of mitochondria. The images show cells before irradiation (left), targets (black points, centre) and the overlay after target acquisition (right) for a representative living cell. Each target point represents a counted number of carbon ions. (**b**) Overview of live irradiation shows snapshots taken from the timelapse video (Video File 1 of the irradiation at SNAKE), whereby irradiation and imaging were performed simultaneously. The micrographs show loss of highly localized mitochondrial signal and a relocalization of TMRE to the cytoplasm (File 1). The white line represents the position of the scanning beam. (**c**) The mitochondrial membrane potential plotted for four selected mitochondria in the representative cell during irradiation with 80 carbon ions per point over time. Four mitochondria were chosen as shown in (**e**) and measured for 10 minutes from start of irradiation. The peaks represent hyperpolarization before depolarization as seen after irradiation. The markers on the graph (E and D) depict the times corresponding to the starting image before irradiation (**e**) and the image after irradiation is complete (**d**). The cytoplasmic background value was measured over the nucleus as labelled with “C” and the coronal measurement area and cell membrane outline are represented in (**d**). The coronal measurement (**c**, lower segment) measured the area around the cell and the increases in signal intensity during and after irradiation as compared to background signal in the same micrograph.

Live cell imaging of mitochondria during irradiation showed a near instant induction of depolarization in mitochondria after targeted application of either protons or carbon ions. The depolarization manifested itself as a highly localized loss of TMRE in the irradiated area only, with no perceptible effect on the rest of the mitochondria in the targeted cell. The induction of depolarization was less than the temporal resolution of the imaging system. When control experiments with the mitochondrial membrane uncoupler FCCP were performed (Fig. 1i) a similar loss of mitochondria specific TMRE signal was seen as after irradiation. A quantification of mitochondrial areas irradiated with 80 carbon ions per point using the standard 6 × 6 matrix shows a highly significant difference between the irradiated and the control mitochondria (Fig. 2). The results show a mean change in fluorescence signal intensity of −87.5 for the irradiated areas and 2.2 in the unirradiated control mitochondria in 20 independently irradiated cells. A loss of fluorescence was not seen when a mitochondrial stain not dependent on ΔΨ_m_, such as Mitotracker green (MTG) (Life Technologies), was used (Fig. 1f). Mitochondrial fluorescence intensity was imaged using time-lapse fluorescent microscopy. When the majority of cells in the field of view were irradiated with counted ions, no more than 10 seconds elapsed between irradiation start and imaging. On the other hand, when imaging and irradiation were performed concurrently (Fig. 3) the time interval between images (300ms) was the limiting factor. The short time interval between initial image acquisition and post irradiation follow-up images ensures minimal movement of mitochondria within the captured frame and allows for the tracking of the irradiated mitochondrial area. The time series acquired from before/after irradiation (Fig. 1) depicted mitochondria just before and 5–10 seconds after targeted irradiation was completed. The micrographs obtained indicate that given a high enough local application of carbon ions or protons, to individual (Fig. 1a,d) or clusters (Fig. 1b,c,e) of mitochondria will induce near instant depolarization under the temporal resolution of the imaging method. The difference in signal intensity of targeted mitochondria is also depicted in pseudocolour images (Fig. 1e,h). The figures show that only the targeted mitochondrial clusters and connected mitochondria undergo a decrease in signal (yellow) after irradiation, compared to rest of the mitochondria and cell (gray). The mitochondria in the irradiated area are depolarized without affecting the overall polarization state of the remaining, unirradiated, mitochondria in the cell during short-term follow-up of up to 30 min.

Given such a pronounced effect, the reproducibility of the depolarization events required a rigorous verification process. To confirm the findings, the experiments were repeated 5 times over the span of two years at SNAKE and then a further two times at the AIFIRA facility. Given the results seen at both SNAKE and AIFIRA using different ions with different LET and that the depolarization was seen with every independent beam setup, the results cannot be classified as an experimental artifact.

### TMRE fluorescence signal and Ψ_m_

To verify that the TMRE molecules accumulated within the mitochondria were not destroyed by the energy deposited by the protons and carbon ions, but relocated within the cell after the highly localized loss of Ψ_m_, an auto targeting routine for all mitochondria was developed. The macro “AutoTarget” detected and applied a radiation spot matrix over all mitochondria in cells exhibiting bright TMRE fluorescence, which then enabled the irradiation of all mitochondria (Fig. 3a). This irradiation point matrix allowed for the targeting of the mitochondria in a cell while sparing the nucleus from damage. The application of ≥80 carbon ions per point yielded total depolarization of all targeted mitochondria and a consequent re-localization of TMRE from a point like mitochondrial staining pattern to a homogeneous whole cell distribution of fluorescence staining (Fig. 3b). When monitoring individual mitochondria over time (Fig. 3c), the fluorescence intensity plot reveals a small dip in fluorescence intensity followed by a peak of hyperpolarization and the final drop of signal indicative of depolarization. After depolarization, the now homogenous cytoplasmic TMRE signal slowly decreased due to the plasma membrane potential of the cell being unchanged and the overall charge of the released TMRE far outweighed that of ΔΨ of the plasma membrane. The plasma membrane potential limits the amount of TMRE which can be present in the cytoplasm due to the fixed membrane potential. Positively charged TMRE dye molecules, whose collective charge is above that of the plasma membrane potential, will be removed to restore charge equilibrium. Therefore, the non-specific, superfluous, positively charged dye from the depolarized mitochondria flows out of the cell again yielding an increased extracellular background signal as measured in a coronal region around the plasma membrane (Fig. 3c). These results clearly indicated that the loss of mitochondrial specific TMRE signal was due to mitochondrial depolarization and not a destruction or bleaching of the TMRE molecules. Further verification of this hypothesis came from experiments at AIFIRA where highly interconnected mitochondria were irradiated. In this case, irradiation of a single interconnected mitochondrial cluster caused all the connected mitochondria in the network to depolarize at the same time (Fig. 1h). Connected mitochondrial signal as far as 18 μm from the edge of the irradiated area showed depolarization within the connected network, again strongly arguing against direct destruction of the TMRE molecules. Thus, the loss of fluorescence intensity is not related to dye destruction or bleaching but to radiation induced mitochondrial depolarization.

### Mitochondrial membrane integrity

At present, the mechanism of the mitochondrial depolarization remains unclear. To test if the targeted irradiation causes mitochondrial membrane rupture, mimicking biological depolarization, membrane integrity was tested. MTG staining was used for non-depolarization related mitochondrial membrane staining. Targeted irradiation of clusters of MTG stained mitochondria showed no significant change in fluorescence signal intensity as was previously seen in the irradiated TMRE labelled cells (Fig. 1f). The lack of change in MTG fluorescence also indicated that the dye was not destroyed in the targeted area by the energy deposited. The MTG experiments were also performed at AIFIRA with the same outcome. To further verify this integrity of the membranes, an additional, non-dye based marker for mitochondrial membrane integrity was used. U2OS cells tagged with RoGFP2 in the mitochondrial matrix were irradiated to test for mitochondrial matrix integrity. As seen in the MTG experiments, the fluorescence signal intensity and localization of RoGFP2 in the mitochondrial matrix stayed constant after irradiation (Fig. 1g). HTB96 U2OS Mito-roGFP2 cells were irradiated at AIFIRA with 6800 protons per point with a matrix of 3 × 3 points. RoGFP2 tagged mitochondria do not show change in fluorescence signal intensity, thus excluding alterations of the mitochondrial matrix composition after irradiation for molecules in the same molecular weight range as roGFP2.

**Figure 4:**
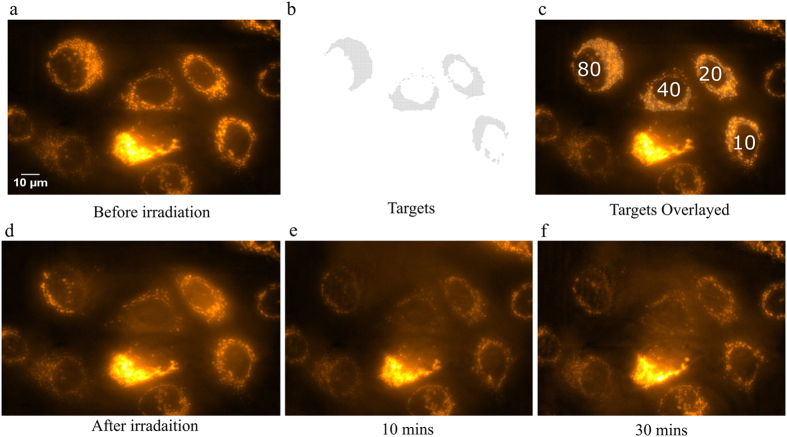
Images from a timelapse imaging sequence performed at SNAKE. (**a**) TMRE (500 nM) stained MCF7 cells in 1 μM PI containing medium for the detection of membrane rupture. (**b**) Targets acquired with “AutoTarget”. (**c**) Cells were sequentially irradiated with 80, 40, 20 and 10 carbon ions per point as labelled. (**d**) Image after irradiation showing TMRE relocalization in the form of intracellular background signal increase is visible, however no PI specific nuclear staining is visible. At 10 min post irradiation (**e**) and 30 min (**f**) there was still no sign of PI specific staining.

As an additional verification of the cellular membrane integrity, 1 μM of propidium iodide (PI) was added to the imaging media during irradiation of A549 and MCF7 cells at AIFIRA and during experiments at SNAKE to check the integrity of the cell membrane after irradiation. Even during irradiation of all mitochondria using the AutoTarget routine in a cell with 80, 40, 20 and 10 carbon ions per point, there was no PI typical nuclear staining (Fig. 4). The lack of PI nuclear staining indicated that the dose of radiation applied was not high enough to rupture the plasma membrane sufficiently to allow PI to enter the cells and stain the DNA up to 30 min after irradiation. The mitochondrial membranes (inner and outer) are estimated to be 22 nm wide^16^; in comparison the cell membrane is estimated to be ~4 nm thick. However the total energy deposited per nanometer of membrane is the same in both cases. The rate of opening and closing of the plasma membrane may however differ from that of the mitochondria which could explain the lack of PI positive cell staining after irradiation.

### Mitochondrial depolarization and dose threshold

**Figure 5:**
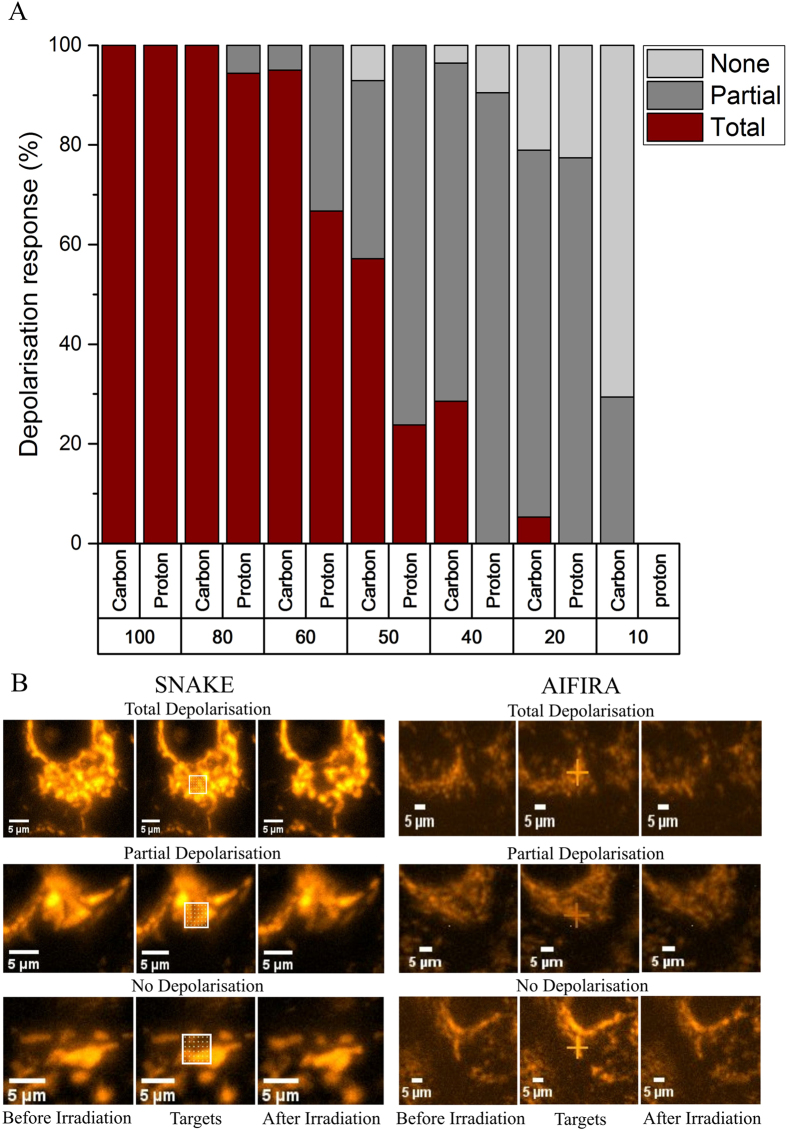
(**A**) Histogram depicting the quantification of polarization state of irradiated mitochondria for experiments performed at SNAKE (carbon ions) and AIFIRA (protons). The percentage of cells featuring Total, Partial or no (None) depolarization are plotted above the number of carbon ions per point (100–10) and equivalent number of protons (3500-350), both decreasing to the right, required to deposit the same amount of energy in a 6 × 6 irradiation point matrix. The number of cells analyzed for each ion application in the order shown are: for carbon 4, 20, 20, 28, 28, 19, 17, and for protons, 19, 18, 12, 21, 21, 30. (**B**) Representative micrographs of total, partial and no depolarization are included for experiments at SNAKE and AIFIRA. Images in the first column depict the cell before irradiation, in the second column, the targets are shown, (6 × 6), and the third column shows the result after irradiation. For total depolarization, micrographs with 100 carbon ions and 3500 protons are depicted; for partial depolarization 40 carbon ions and 1500 protons; and for no depolarization 20 carbon and 700 protons are depicted.

A small matrix of irradiation spots (6 × 6 points, 1 μm between each point), or a whole cell irradiation matrix, showed that given a high enough number of ions (carbon or equivalent number of protons) per point, the dose deposition yielded a total depolarization in the targeted mitochondria. A 6 × 6 irradiation point matrix was used to compare the effects of varying ion numbers on mitochondrial depolarization. Between 100 and 80 carbon ions per point, total depolarization of the targeted area was observed for carbon ions. One hundred carbon ions or equivalent protons caused total depolarization in 100% of the irradiated areas. When 80 carbon ions were applied, total depolarization was seen in 100% of the targeted areas and in 95% of the irradiated areas for protons (Fig. 5B). Below 80 ions, a larger range of responses were observed in the Ψ_m_. A threshold below which total mitochondrial depolarization was not seen was determined to be ~10 carbon ions per point or equivalent protons. Between 20 and 100 carbon ions the degree of depolarization varied from a partial effect (flickering or short term loss of potential) to total depolarization. The most interesting depolarization phenomena were seen in the intermediate range, some mitochondria only partially depolarized, flashed or depolarized totally and reappeared with less intensity within seconds of the targeted irradiation. These effects were labelled as “partial depolarization” and along with total and absence of depolarization are summarized for both carbon ions and protons in Fig. 5.

The lower the dose per point, the more likely it was that the mitochondria would not totally depolarize or in fact show no overall loss of signal at all, indicating that the energy deposited was not sufficient to induce depolarization. The differences between the responses of the mitochondria to carbon ions and the equivalent number of protons (Fig. 5A,B) required for the same energy deposition showed the same overall trend but differ slightly in their overall effect, which may be due to differences in beam spot size and the manner and precision in which the ions are counted and applied. In addition, the LET of the particles must be taken into account, as the LET of the particles is not the same and therefore the relative biological effect cannot automatically be assumed to be the same either.

## Discussion

For the first time we have shown that highly localized targeted mitochondrial irradiation using 55 MeV carbon ions and 3 MeV protons induces mitochondrial membrane potential loss. The number of ions required to induce instant mitochondrial membrane depolarization is rather high: the results indicate that 80 carbon ions or the equivalent number of protons per point (same equivalent energy) focused to a single point or in a matrix of points are required for near instant and total depolarization of all targeted mitochondria. Below this applied dose of ions, mitochondrial depolarization is not binary in its nature since a partial reduction in the TMRE signal is observed, as well as mitochondrial flashes. When the mitochondrial loss of TMRE was quantified (Fig. 2) a large and significant (p < 0.001) difference was seen between unirradiated and irradiated samples. The effect size, as calculated by Cohen’s d produced a value of 3.85, further verifying the highly significant difference between control and irradiated groups means. The total changes of gray values depicted are also within the same region as the results from the FCCP control experiments (Fig. 2) performed using the same microscope setup and parameters The time required for application of 80 ions is well below that of the imaging setup, so a progressive loss of Ψ_m_ stepwise as each ion traverses the mitochondria is not observable. At the threshold of 10 carbon ions per point there is little to no visible effect of any of the above mentioned phenomena anymore. In comparison to previous experiments using lasers, heavy ion irradiation allows for a calculation of deposited energy at any point along the particle’s track. This is a great advantage over laser irradiation as it allows for a far more accurate assessment of the energy required to induce a biological effect. Laser irradiations performed by Yoon *et al*. show plasma membrane rupture (PI, DAPI) and apoptosis between 6–25 μJ within minutes^5^. In comparison, the results shown here enable a precise deposition of energy within the range of *pico* to *femto* Joule, depending on size of mitochondria targeted, which is evidently sufficient to depolarize individual mitochondria without disrupting the plasma membrane, as has been confirmed by lack of PI signal up to 30 minutes after irradiation (Fig. 4).

The differences in the mitochondrial responses between carbon ions and protons seen in Fig. 5A may well be attributed to a difference in the accuracy of counting of the ions. The proton irradiation at AIFIRA relies on the count rate of the ions coming out of the accelerator and is checked multiple times per experiment. In comparison carbon ions at SNAKE are individually counted, ensuring a highly accurate number of ions applied. A drop in count rate of protons during the irradiation could therefore be the explanation for the difference in the responses seen in Fig. 5 for protons compared to carbon ions. The overall mitochondrial response to protons and carbons, however, seems to be very similar and the total energy deposited seems to be a good guideline for mitochondrial depolarization as the effect is seen after irradiation with both ion types.

Loss of mitochondrial membrane potential, as confirmed by the relocalization of TMRE from the mitochondria to the cytoplasm (Fig. 3b and Video File 1) and then to the extracellular space, as measured in the coronal region (Fig. 3c), indicates a distinct change in mitochondrial membrane potential. In detail, a small dip in fluorescence signal followed by a spike in intensity, again followed by the strong depolarization reaction, shows an interesting dynamic in mitochondrial depolarization-polarization and demonstrates a state of complete depolarization that cannot yet be modelled. When looking at a longer time scale of up to 600 s after irradiation, there is an additional dynamic in repolarization and depolarization of the individual irradiated mitochondria, but also within the cytoplasm and even in the coronal region. The correlated increase of cytoplasmic signal with the depolarization of the mitochondria reveals the redistribution of TMRE and thus the changes within the cell. Furthermore the TMRE released from the irradiated mitochondria is taken up by the surrounding mitochondria, leading to an increased peak before the radiation induced loss of TMRE (Fig. 3c and Video File 1). If TMRE would have been destroyed or inactivated by the irradiation, such a relocalization and uptake by neighboring mitochondria would not occur as shown in mitochondria labelled 4 (Fig. 3c). This dynamic relocalization of the TMRE from the irradiated mitochondria indicates that TMRE is not destroyed or inactivated by the irradiation.

The mechanism of this membrane potential change is currently unclear, however the MTG (Fig. 1f) and PI results (Fig. 4) are an initial indication that the TMRE relocalization may not necessarily be based on irreversible physical membrane rupture. Although indicative, the lack of change in roGFP2 and MTG fluorescence intensity cannot completely rule out subtle changes in membrane integrity. Carbon ion and proton irradiation with up to 100 ions per point would deposit 1.5 keV (~0.24 fJ) into the plasma membrane, after which the plasma membrane permeability remained unchanged in relation to PI uptake. The mitochondrial membrane structure (inner, inter membrane space and outer) are 5.5x thicker than the cell membrane but absorb the same amount of energy per nanometer of membrane. So the closing and opening of the membranes may well play a more crucial role than the thickness alone. After irradiation, a distinct and total change in membrane potential was observed, which in the cases of higher ion/point applications remained depolarized. The indication here could be that changes in the membrane structure, such as lipid peroxidation, may have occurred which has in the past been shown to cause changes in membrane permeability^17^. The structural changes induced by these types of radiation may also be so small and distinct that they are not large enough to allow PI to traverse the membrane. The formation of radiation induced transient nanopores cannot be ruled out at this stage either. Mitochondrial membrane transition pore opening is also an unlikely cause of the depolarization, as initial experiments with a transition pore inhibitor CyclosporinA show the same instant and total depolarization of irradiated mitochondria. In addition to the structural changes induced by direct interaction, radiation induced reactive oxygen species which are formed after the ions interact and ionize water molecules, may play a role in the process of depolarization. Radiation induced reactive oxygen species (ROS) are known to cause changes in mitochondrial membrane potential, however further work is required to directly link radiation induced ROS at the site of targeted irradiation to the depolarization seen in this work. The damage to membranes induced by transient ROS formation could be a cause for this depolarization. Furthermore, the secondary electrons created in the path of the ions by ionizations may lead to a highly localized disruption of the electron transport chain within the mitochondria, which, in turn could also be the cause of the loss of the membrane potential.

This body of work describes a novel method for single mitochondrial manipulation and monitoring *in situ* by precisely controlled energy deposition, and opens up the field for further in-depth analysis. The results show a previously unseen change in mitochondrial membrane potential as indicated by loss of mitochondrial TMRE fluorescence, the mechanism of which still remains to be elucidated. To further assess this radiation induced loss of membrane potential, new methods will have to be devised and their limitations will have to be overcome, but the results will have the ability to shed new light not only on mitochondria’s response to radiation but also a mechanism of radiation induced depolarization.

## Materials and Methods

### Cell culture and mitochondrial staining

MCF-7 breast adenocarcinoma cells (ATCC HTB22) and A549 lung carcinoma cells (ATCC CCL-185) were grown in DMEM (D6429, Sigma Aldrich) completed with 10% v/v FCS (Sigma) and 100 mg.mL^−1^ penicillin/streptomycin (Sigma Aldrich) at 37 °C, 95% humidity and 5% CO_2_ saturated atmosphere. HTB U2OS were grown in McCoy’s medium (Dutscher, L0211-500) completed with 2 mM L-glutamine, 100 mg.mL^−1^ penicillin/streptomycin (Thermo Fischer) and with 10% v/v FCS (Dutscher). HTB96 U2OS cells were stable transfected with the Matrix-roGFP2 constructs (Plasmid #49437, Addgene). This plasmid expresses the thiol redox‐sensitive ratiometric fluorescent sensor roGFP2 in the mitochondrial matrix, under the control of the CMV promoter. Viromer Red transfection reagent was used for transfections in accordance with the manufacturer’s protocols. The transfection efficiency was 80–90% in all experiments.

Twelve hours before irradiation, cells were plated into the custom designed live cell imaging containers^18^ and allowed to adhere to the scintillator surface (SNAKE) or polypropylene foil^19^ (AIFIRA), which had previously been treated with Corning CellTak (as per manufacturer’s instructions). Thirty minutes before irradiation, pre-warmed DMEM medium containing 25 mM HEPES buffer and 250 nM TMRE (Enzo Lifesciences), or 200 nM Mitotracker green FM (Life Technologies) for the control experiments, were added to the cells in the dark and incubated as above. The TMRE in medium was left on the cells for 30 min so that the mitochondria could reach an equilibrium of TMRE uptake. The media containing TMRE was then removed and pre-warmed media with an additional 25 mM HEPES was added to the sample before being placed into the heated microscope table at SNAKE and AIFIRA. To verify plasma membrane integrity during experiments, 1 μM Propidium iodide (PI) was added to the medium during imaging and irradiation. As a control for PI staining upon membrane rupture, 10% (v/v) Triton X-100 (Sigma Aldrich) was added to cells in PI containing medium to disrupt the plasma membrane. As a control for depolarization level, 1 μM FCCP (Enzo Lifesciences) was added to MCF7 cells incubated with TMRE to show the effect of total uncoupling of the mitochondrial membrane potential under the same imaging conditions.

### Microbeam irradiation and live cell imaging at SNAKE

Irradiation was performed by spot application of counted, individual carbon ions with initial beam energy in vacuum of 55 MeV, as previously described by Siebenwirth *et al*.^20^. After leaving the vacuum, the ions lost about 10 MeV of energy by penetrating the beam exit window and approximately 20 μm of culture medium resulting in an LET in water of 350 keV/μm at the cells. Samples were imaged using a Zeiss Axiovert 200 M with a 63x Objective (LCI Plan-Neofluar 63x/1.3 Ph3 Imm Corr M27, Zeiss) and a Colibri LED light source (Zeiss). The microscope is tilted by 90° so that it is in line with the beam exit nozzle as described by Hable *et al*.^18^. For excitation of the TMRE, a 555 nm LED was used at 2–4% intensity and an appropriate filter cube (43 HE Zeiss) with varying exposure times depending on the overall fluorescence of the given area being imaged. A maximum exposure time of 500 ms was never exceeded. The images were captured using a Zeiss MRm rev. 3 CCD.

To detect and irradiate all mitochondria in the targeted cell a Visual Basic macro “AutoTarget” was written and integrated into Axiovision. The macro overlayed an irradiation target matrix with a defined distance of 0.5 μm between irradiation points over mitochondria exhibiting bright fluorescent signal. The mitochondria were automatically selected by thresholding, leaving the nucleus, which was lacking fluorescence signal, devoid of targets and therefore spared of dose. The density of irradiation points was therefore 4 per μm^2^.

### Microbeam irradiation and live cell imaging at AIFIRA

Irradiation was performed by spot application of 3 MeV protons (LET in water of 10 keV/μm), as previously described by Bourret *et al*.^19^. Sample irradiation/imaging was performed at 37 °C and was limited to a maximum of 2 hours per sample to ensure constant conditions for the cells within the sample holder. Samples were imaged using a Zeiss AxioObserver Z1 with a 63x lens (LD Plan-Neofluar, NA 0.75). For excitation the same 555 nm LED and 43 HE filter cube were used as in Munich and for image acquisition a Zeiss CCD (Axiocam Mrm rev 03) was used. Similar imaging conditions and equivalent CCD settings were maintained as closely as possible between both institutions apart from binning which was adjusted to 2 × 2.

All analysis of micrographs was performed using FIJI (Fiji Is Just ImageJ) and plotted with Origin pro plotting software.

### Statistical analysis

To analyze the statistical significance of the quantification of the micrographs a two-tailed students T-test for paired samples was performed. In addition, the equation to calculate Cohens d (Eq. 1) was used to determine effect size for the sample set.

d is the Cohen’s d value, μ_1_ is the mean of the irradiated population, μ_2_ is the mean of the control (unirradiated) population and *σ*_*pooled*_ is the pooled standard deviation of both of the samples and is calculated by Eq. 2


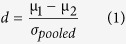



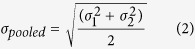


## Additional Information

**How to cite this article**: Walsh, D. W. M. *et al*. Live cell imaging of mitochondria following targeted irradiation *in situ* reveals rapid and highly localized loss of membrane potential. *Sci. Rep.* **7**, 46684; doi: 10.1038/srep46684 (2017).

**Publisher's note:** Springer Nature remains neutral with regard to jurisdictional claims in published maps and institutional affiliations.

## Supplementary Material

Supplementary Information for Video

Supplementary Video
