# Supplementary material for: Live cell imaging of mitochondria following targeted irradiation in situ reveals rapid and highly localized loss of membrane potential
Source: Sci Rep. 2017 Apr 25;7:46684. doi: 10.1038/srep46684 (PMC5404225; doi:10.1038/srep46684)
Supplement: Supplementary Information for Video [file srep46684-s1.doc]

Live cell imaging of mitochondria following targeted irradiation in situ reveals rapid and highly localized loss of membrane potential

Dietrich W. M. Walsh* ^1,2^, Christian Siebenwirth ^1,2^, Christoph Greubel ^1^, Katarina Ilicic ^2^, Judith Reindl ^1^, Stefanie Girst ^1^, Giovanna Muggiolu ^3,4^, Marina Simon ^3,4^, Philippe Barberet ^3,4^, Hervé Seznec ^3,4^, Hans Zischka ^5^, Gabriele Multhoff ^2^, Thomas E. Schmid ^2^, Guenther Dollinger ^1^

*1 Universität der Bundeswehr München, Institut für Angewandte Physik und Messtechnik, D-85577 Neubiberg, Germany*

*2 Klinikum rechts der Isar, Technische Universität München, D-81675 München, Germany*

*3 Université de Bordeaux, Centre d’Etudes Nucléaires de Bordeaux Gradignan (CENBG), Chemin du Solarium, 33175 Gradignan, France*

*4 IN2P3, CNRS, UMR5797, Centre d’Etudes Nucléaires de Bordeaux Gradignan (CENBG), Chemin du Solarium, 33175 Gradignan, France*

*5 Institute of Molecular Toxicology and Pharmacology, Helmholtz Center Munich, German Research Center for Environmental Health, D-85764 Neuherberg, Germany.*

*Corresponding Author: [dietrich.walsh@unibw.de](mailto:dietrich.walsh@unibw.de)

**Video File 1**: Targeted irradiation of Mitochondria with “AutoTarget” macro at SNAKE. All mitochondria were automatically targeted using the “AutoTarget” thresholding macro as described, each target was then irradiated with 80 carbon ions. Imaging and irradiation were performed concurrently.
